# Foreign Medical Matters

**Published:** 1888-04

**Authors:** 


### FOREIGN.

### MEDICAL MATTERS.

MEDICAL EDUCATION AND WOMEN IN INDIA—A correspondent, writing to the *Journal and Examiner* from Pullumpett, Cuddapah District, Madras Presidency, India, says:

There are not many female surgeons and practitioners in this benighted Presidency. Women are not so much advanced in civilization as in the British countries. In America females are holding very prominent and useful positions in life. In India the state of things is quite different. The education of women is at a very low ebb, and liberty is a thing quite strange to them. The superstition and prejudice that have been reigning in India for the many dark ages are great barriers to the march of Western civilization. Thanks to the Almighty Providence who has vouchsafed to us the blessings of Western culture and freedom.

Only two native females have lately obtained the qualification for medical practitioners by passing the examination prescribed by the Government of Madras, and they have been lately appointed to take charge of the hospitals for women. Mostly, the practitioners, like myself, are males. There are a few ladies from America, connected with the American Board of Foreign Missions, who are doing very good and noble work among the Eastern sisters in this Presidency. I am in charge of a dispensary where out-patients are to be treated for their several complaints. I have obtained the diploma of apothecary by passing the required examination, after a course of study extending over four years in the Madras Medical College.

MEDICAL PRACTICE IN CHINA.—A writer in a letter from Kinngchow, Hainan, China, May 21, 1887, says: Ever since I came to Hainan I have seen more or less of the medical work of this station, and have always found it full of interest. Last year after May 1, our two doctors treated over 12,000 (or rather received over 12,000 visits from out-patients), besides treating 200 in-patients, and performing nearly a thousand surgical operations. The number coming was so great at times that they could not all be seen in one day, and the press to get into the dispensing room, even in spite of the efforts of our helpers, was so great, that at times the weak were in danger.

Those seeking treatment suffer mostly from skin diseases and from fevers. Blindness is quite common here, and sight has been restored to several by the removal of cataract, and several cancers have been removed. There is no lack of patients, and no one knows how much direct good is done by the removal of suffering; but the indirect good is still more incalculable. As one instance of indirect good resulting to us from the medical work, we are enabled to remain in this city during the examinations, with no other annoyance than the curiosity which a foreigner is sure to excite in any part of China.

The number coming from all directions, and often several days' journey for medicines, present an interesting congregation.

---

JOPPA MEDICAL COLLEGE.—An extract from the report of a hospital connected with the Joppa (*Jaffa*) Medical Mission, Palestine, says: The Medical Mission is carried on five days in every week, the patients often beginning to gather round the gate as early as 6 a. m., in their eagerness for the nine o'clock opening. The total number of attendants from November 1, 1885, to December 31, 1886, was 11,176. During the same period, notwithstanding the trials and hindrances of the work, 231 patients have been nursed in the hospital, of whom twelve have died, seven being admitted in a hopeless condition. Of these in-patients, eight were Jews, ten were Maronites, three Latins, six Protestants, nineteen Greeks, one Armenian, one Copt, and 183 Moslems. The increased accommodation of the new hospital has admitted of a ward being set apart for women, already occupied by five patients.

---

GRADUATES FROM FRENCH MEDICAL SCHOOLS.—The number of Doctors of Medicine graduated by French faculties during the past five years is as follows: 1882–83, 622; 1883–84, 590; 1884–85, 575; 1885–86, 546; 1886–87, 624. The sudden increase in 1886–87 was due to the new law requiring medical men of the navy to take the degree from one of the six faculties.

STUDENTS IN PARIS IN 1886–87.—On October 15 there were 3,966 medical students registered in Paris, and 582 graduates. Of the latter, 479 were French, and 103 foreign. Among the foreign students the Americans and Servians headed the list with 20 each. Of the 11 women students registered, 10 were Russian and one Greek.

---

THE JAPANESE SANITARY ASSOCIATION, which has 4,700 members, was founded in 1883, and now has twenty-eight branches in different parts of Japan. At the annual meeting of the association in Tokio, in May, 1887, there were 20,000 visitors to the hygienic exhibition. The name of the official journal of the association is *Dai Nippon Shiritsu Eiseikwai Zasshi.*

---

Two thousand eight hundred and seventy-nine patients were treated last year at the Peking, China, Medical Mission Hospital, and at the New Dispensary at Nanking over 250 a day were prescribed for.

---

DAMASCUS MEDICAL MISSION.—Dr. Mackinnon reports regarding his medical missionary work at Damascus, that during the first eighteen months of his service in that city he treated more than 2,000 cases.

